# Genomics of Brain Disorders 4.0

**DOI:** 10.3390/ijms25073667

**Published:** 2024-03-25

**Authors:** Ramón Cacabelos

**Affiliations:** International Center of Neuroscience and Genomic Medicine, EuroEspes Biomedical Research Center, 15165 Bergondo, Spain; rcacabelos@euroespes.com

## 1. Introduction

Several historic, scientific events have occurred in the decade 2013–2023, in particular the COVID-19 pandemic. This massive pathogenic threat, which has affected the world’s population, has had a devastating effect on scientific production worldwide. Repercussions on people with cardiovascular diseases (25–30%), cancers (20–25%) and brain disorders (10–15%) throughout the world represent more than 65% of the global morbidity and mortality data (>80% in developed countries). In the same decade, there has been a spectacular explosion in research on structural and functional genomics, epigenetics, transcriptomics, proteomics, and metabolomics, laying the foundations for precision medicine (genomic and personalized medicine). There have been large impacts on the following three key medical areas: knowledge of the pathogenesis of diseases; molecular diagnostics (predictive biomarkers, which facilitate early diagnosis, even in the asymptomatic phases); and pharmacogenomics, allowing us to personalize pharmacological treatments to increase the efficacy of medicines, minimize the undesirable effects, and reduce the costs. The impact of genomics on medical practice is reflected in the number of publications published in this decade, although there is a monumental gap between the numbers of general publications on a given pathology and those referring to the genomics of a specific disease. 

## 2. Cardiovascular Disease

The main cardiovascular diseases (coronary heart disease, 200 million; peripheral artery disease, 110 million; stroke, 100 million; atrial fibrillation, 60 million) affect almost 500 million people in the world, constituting the leading cause of death (around 18 million deaths/year), with a cost of more than USD320 billion in the USA alone [1,2,3].

Upon analyzing the number of publications referenced in the NCBI’s PubMed database, we found that the number of generalist publications grew by 3%/decade (from 99,765 in 2013 to 103,575 in 2023, with a total of 931,704 publications/decade). On the other hand, the number of publications on the genomics of cardiovascular disorders grew by 58% (from 2577 in 2013 to 4076 in 2023 out of a total of 32,451 publications/decade). 

## 3. Cancer

There are an estimated 18.1 million cancer cases/year throughout the world (9.3 million men and 8.8 million women). Breast (12.5%) and lung cancers (12.2%), followed by colon cancer (10.7%), are the most common forms of cancer in the world. In the U.S. alone, the cost of cancer amounts to more than USD210 billion/year [4,5].

The number of generalist publications on cancer grew by 53% globally (from 171,754 publications in 2013 to 264,240 in 2023 out of 2,055,272 publications/decade). At the same time, the number of studies carried out on cancer genomics grew by 68% (from 15,429 in 2013 to 26,041 in 2023 out of 214,055 publications/decade).

## 4. Neuroscience vs. Genomics

From 2013 to 2023, the two fields of knowledge that expanded the most were neuroscience and genomics. The number of publications related to neurosciences grew by 168% (from 21,011 in 2013 to 56,425 in 2023 out of a total of 418,762 publications/decade), and the number of those on genomics grew by 31% (from 74,481 to 97,601 out of a total of 838,383 publications/decade).

## 5. Impact of COVID-19

With the outbreak of the COVID-19 pandemic and the consequent general halt in global scientific activities, universities, research centers, and the pharmaceutical industry suffered. Many projects were paralyzed, laboratories were closed, and funds were disinvested in science, all of which drastically impacted global scientific production. In all areas, without exception, there was an uninterrupted growth in scientific output until 2021, which is when research from 2020 was published; however, after 2021, there were general drops in scientific production of 17% in relation to cardiovascular diseases (from 124,432 in 2021 to 103,575 publications in 2023), 25% in relation to cancer (284,850 in 2021 to 264,240 in 2023), 11% in relation to diseases of the nervous system (87,579 in 2021 to 78,215 in 2023), 5% in relation to neurosciences (61,158 in 2021 to 56,425 in 2023), and 7% in relation to genomics (110,981 in 2021 to 97,601 in 2023). This average drop of 5–8% across all disciplines could not even be offset by the explosion of publications on COVID that appeared in 2020 (93,909), 2021 (140,786), 2022 (130,189), and 2023 (88,178), with a decrease of 8% between 2021 and 2022 and a drastic reduction of 38% between 2022 and 2023. 

## 6. Genuine Behavior of Brain Diseases

The various diseases of the nervous system, despite their plurality and heterogeneity, share some common determinants. More than 80% of brain pathologies have a genetic component, whereby genomic dysfunction is key for the disease to manifest with a determined phenotypic profile. The convergence of genomic, cerebrovascular, and environmental factors characterizes the phenotype of each pathogenic process. Another important aspect to consider is that encephalopathies in adult and elderly patients destroy the brain decades before they show symptoms; for this reason, it is essential to identify the populations at risk, to prevent and delay their expression or, in the best case, avoid their clinical manifestation through genomic screening and epigenetic fingerprinting.

In epidemiological terms, in this decade, 970 million people suffered from some type of mental disorder annually, with depression and anxiety as the most prevalent pathologies [6]. The average cost of mental illness in Europe is close to USD800 billion [7], and the cost of the more than 50 million Americans (21.8% of the population) who suffer from a brain problem is USD1.5 trillion [8]. 

The global incidence of neurological diseases is set at 10,259.50 per 100,000 inhabitants, with a clear increase in the incidences of neurodegenerative diseases (Alzheimer’s and Parkinson’s), epilepsy, headaches, and bipolar disorder from 1990 to 2019. By region, North America and Europe present the higher increase in brain disorder cases [9,10].

In this decade, neurological disorders have been the leading cause of disability and the second leading cause of death. The main causes of disability were stroke (42%), migraines (16%), dementia (10%), and meningitis (116%) [10]. 

Migraines are one of the most common ailments in the world, with a high prevalence in Europe and low prevalence in Africa. The average prevalence of migraines in the world is 15%, with an increase of almost 2% from 1990 to 2020, to a total of 1.1 billion cases/year (from 8,277 to 22,400.6 cases per 100,000 inhabitants) and an economic burden of more than USD11,000 patient/year in the USA [11,12,13,14].

Some 6.7 million Americans suffer from dementia today, and it is estimated that this figure could rise to 13.8 million by 2060 if no effective treatment or prevention formula is developed in the coming years. There could be more than 70 million people affected in the world right now, making Alzheimer’s disease the fifth leading cause of death. In the last 25 years, the number of deaths caused by Alzheimer’s has increased by 145%. The total cost of dementia in people over 65 years of age is USD345 billion in the USA [15,16]. The cost of dementia exceeds USD800 billion worldwide (>1% of GDP), with an average cost per patient/year fluctuating between USD30,000 and USD60,000, depending on the country, social status, quality of medical care, and stage of the disease. Approximately 20% of the direct costs are associated with pharmacological treatment, with little cost-effectiveness. Alzheimer’s disease is the most prevalent form of dementia (50–60%), followed by vascular dementia (30–40%), other forms of dementia (10–15%), and mixed dementia, which is the most prevalent form of dementia (>70%) in patients over 75 years of age [17]. 

Globally, an estimated 280 million (3.8%) people suffer from depression (5% of adults and 5.7% of those over 65 years of age), with 34% of cases reporting symptoms of depression [18].

Despite these frightening figures regarding the main diseases of the brain, scientific production throughout the world is very irregular. The three most-studied pathologies with the highest pharmaceutical investment are depression, with 275,681 publications/decade and a growth of 71% (23,266 generalist studies in 2013 and 39,993 in 2023); anxiety, with 180,492 publications per decade and an increase of 112% from 2013 (13,267) to 2023 (28,950); and Alzheimer’s disease, with 113,227 publications/decade and a 94% increase from 2013 (8773) to 2023 (17,057). These are followed by Parkinson’s disease, with 74,213 publications/decade (an increase of 64% from 2013 (6081) to 2023 (10,024)); epilepsy (71,148 publications/decade), with an increase of 40% from 2013 (6412) to 2023 (9066); schizophrenia (57,483 publications/decade), with no increase (0.1%) from 2013 (6581) to 2023 (6636); multiple sclerosis (52,315 publications/decade), with an increase of 35% from 2013 (4757) to 2023 (6468); autism (50,109 publications/decade), with an increase of 113% from 2013 (3524) to 2023 (7527); and migraines (19,785 publications/decade), with an increase of 35% from 2013 (1780) to 2023 (2731).

## 7. Increase in Number of Genomic Studies

If we look at publications related to the genomics of brain diseases, the number and nature of publications are more irregular and heterogeneous. In this case, Alzheimer’s disease accounts for the highest number of publications per decade (8995), with an increase of 149% from 2013 (563) to 2023 (1405). Alzheimer’s is followed by depression, with 6723 publications/decade and an increase of 95% from 2013 (501) to 2023 (979); autism (6901 publications/decade), with an increase of 102% (from 347 in 2013 to 704 in 2023); schizophrenia (5309 publications/decade), with an increase of 24% (from 509 in 2013 to 634 in 2023); Parkinson’s disease (4637 publications/decade), with an increase of 66% (359 in 2013 to 599 in 2023); epilepsy (4383 publications/decade), with an increase of 109% (from 306 in 2013 to 640 in 2023); multiple sclerosis (2646 publications/decade), with an increase of 18% (from 261 in 2013 to 3110 in 2023); anxiety (2080 publications/decade), with an increase of 125% (from 140 in 2013 to 315 in 2023); and migraines (1133 publications/decade), with an increase of 105% (from 52 in 2013 to 107 in 2023). 

The potential effect of COVID-19 on scientific output related to the genomics of brain diseases between 2021 and 2023 was also uneven for each disease. The quantity of scientific output fell for autism (4%), depression (6%), Parkinson’s (19%), and multiple sclerosis (10%), and it rose for schizophrenia (0.3%), anxiety (1%), Alzheimer’s (3%), migraines (28%), and epilepsy (1%). According to these data, COVID-10 had a greater influence on the number of generalist publications than those related to genomics. 

From a strictly scientific point of view, in terms of quality, genomics contributed enormously to a better understanding of the etiopathogenesis of diseases of the nervous system [19,20,21,22,23,24,25,26,27,28,29,30,31,32], to the discovery of diagnostic biomarkers [33,34,35], and to accelerating the implementation of new pharmacogenomics protocols, both for the use of common drugs and for the development of new drugs [17,36,37]. 

This Special Issue, the fourth edition of the Genomics of Brain Disorders, is a humble example of the different aspects that can be covered by the study of genomics in various diseases of the nervous system. In my capacity as the Guest Editor, I would like to thank the *International Journal of Molecular Sciences* for its kind invitation, and I would like to express my sincere gratitude to all the authors who have participated in this and the three previous editions for their excellent work and extraordinary collaboration.
